# Water hydrogen uptake in biomolecules detected via nuclear magnetic phosphorescence

**DOI:** 10.1038/s41598-019-53558-8

**Published:** 2019-11-19

**Authors:** Aude Sadet, Cristina Stavarache, Florin Teleanu, Paul R. Vasos

**Affiliations:** 1 Research Institute of the University of Bucharest (ICUB), 36-46 B-dul M. Kogalniceanu, RO-050107, Bucharest, Romania; 2 “C. D. Nenitescu” Centre of Organic Chemistry, 202-B Spl. Independentei, RO-060023, Bucharest, Romania; 3 Faculty of Chemistry and Chemical Engineering, Babes-Bolyai University, Arany Janos 11, Cluj-Napoca, Romania; 4 “Horia Hulubei” National Institute for Physics and Nuclear Engineering IFIN-HH, Extreme Light Infrastructure - Nuclear Physics ELI-NP, 30 Reactorului Street, RO-077125, Bucharest-Magurele, Romania

**Keywords:** Peptides, Biological physics

## Abstract

We introduce a new symmetry-based method for structural investigations of areas surrounding water-exchanging hydrogens in biomolecules by liquid-state nuclear magnetic resonance spectroscopy. Native structures of peptides and proteins can be solved by NMR with fair resolution, with the notable exception of labile hydrogen sites. The reason why biomolecular structures often remain elusive around exchangeable protons is that the dynamics of their exchange with the solvent hampers the observation of their signals. The new spectroscopic method we report allows to locate water-originating hydrogens in peptides and proteins via their effect on nuclear magnetic transitions similar to electronic phosphorescence, long-lived coherences. The sign of long-lived coherences excited in coupled protons can be switched by the experimenter. The different effect of water-exchanging hydrogens on long-lived coherences with opposed signs allows to pinpoint the position of these labile hydrogen atoms in the molecular framework of peptides and proteins.

## Introduction

New spectroscopic probes for exchangeable hydrogens in biomolecules are needed, as water-exchanging hydrogens often remain elusive for spectroscopy, and, consequently, for structural biology in the liquid state. Biomolecular uptake of water protons is essential for cell homeostasis and for cell function. The role of water hydrogen atoms in the mechanisms that set the tempo of living systems is reconsidered as new insights are gained on water interactions with biomolecules: hydrogens in water are messengers in enzymatic function^1^ and main players in cancer radiotherapy via radiolysis^2^. Atomic and molecular spectroscopies explore the interactions of water-originating protons with biomolecules within the bounds set by the intrinsic resolution and sensitivity of each analytical method^3–5^. Fluorescent or phosphorescent probes are used to observe water interactions occurring, e.g., upon protein unfolding. The long lifetimes afforded by singlet-triplet transitions in phosphorescent probes, which can be used for medical imaging, are known to be sensitive to inter-molecular interactions^6,7^. However, most chromophores are difficult to introduce in molecular folds without perturbing the structure. Atomic resolution in liquid state can be attained by Nuclear Magnetic Resonance (NMR). In high magnetic fields, NMR is particularly adapted to follow water molecules and their interactions with peptides or proteins. Ensemble structural information including water in solvated structures can be obtained by 2D and higher-dimension NMR spectroscopy^8^. The interactions of water with proteins and small molecules are used in NMR-based pharmaceutical drug screening^9^. Fundamental studies of the magnetic interactions between water and biomolecules have shown wide-reaching practical implications in clinical imaging: water-proton magnetisation transfer to nuclear spins in endogenous molecules is at the basis of a widely-used contrast mechanism, CEST-MRI^10^. The limit for sensitivity to water interactions with other molecules is set by the magnitude of magnetic dipoles, the statistics of their reciprocal orientations, and their motions^11^. New methods for detecting water-exchanging protons by NMR can be obtained by changing the observed magnetic dipoles. The magnitude of the involved dipoles can be, for instance, enhanced by the use of electrons instead of nuclei, as in Paramagnetic Relaxation Enhancement^12^.

Herein, we present another way of modifying the observed nuclear magnetic moments and using the modification to study interactions: grouping two or several magnets together, i.e., in paired or collective nuclear states^13–15^. For two *J*-coupled nuclei, the populations of nuclear singlet trickle slowly to nuclear triplet states. This is a transition from spin-exchange antisymmetric states to symmetric ones. Singlet-based spectroscopy is to standard NMR what phosphorescence is to fluorescence in electronic spectroscopy. The observation of singlet states relies on slow-paced transitions that occur when a change of spin-permutation symmetry is involved. Their population provides extended memory for nuclear spin order. Slowly-relaxing nuclear transitions for nuclei or groups of nuclei featuring low dipolar interactions are adapted for use in hyperpolarised endogenous molecules as magnetic resonances tracers *in vivo*^16–18^. These molecular biomarkers detect the rates of metabolic processes such as glucose metabolism at different endpoints^19^, harvesting functional information for medical imaging in a non-invasive manner. Transitions between nuclear singlet and triplet configurations were first observed as low-frequency oscillations in low magnetic fields^20^. In high magnetic fields, these transitions improve spectral resolution, as singlet-triplet long-lived coherences (LLC’s)^21^ feature decays up to 9 times slower than those of standard NMR transitions, yielding a proportional narrowing of the observed spectroscopic lines. The relaxation behaviour of LLC can be used for imaging^22^ or to improve contrast in spectra of complex chemical mixtures, as already demonstrated for long-lived states^23^.

We report in this manuscript for the first time on the sensitivity of LLC-related states to biomolecular structure, via the permutation symmetry of the state. We have encoded LLC’s with different signs on naturally-occurring amino-acids in peptides and proteins and observed that their interaction with water-exchanging hydrogens yields a new way to establish the structural position of the latter.

## Results and Discussion

**Figure 1:**
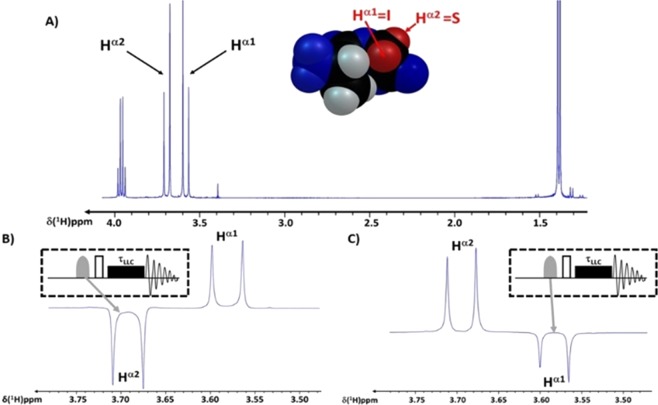
Preparation of LLC’s on AlaGly dipeptide. (**A**) ^1^H spectrum of AlaGly. The signal of Ala-H^α^ is a quadruplet (^3^*J*_*Ala-H*α*/Ala-CH3*_ = 7.1 Hz) at 3.96 ppm, Gly-H^α1,2^ signals are doublets (^2^*J*_*IS*_ = 17.2 Hz) at 3.58 and 3.69 ppm, respectively, and the Ala-CH_3_ signal is a doublet (^3^*J*_*Ala-H*α*/Ala-CH3*_ = 7.1 Hz) at 1.39 ppm. I and S indicate inequivalent protons H^α1^ and H^α2^, respectively. Inset: spacefill representation of AlaGly, with positions containing water-exchangeable groups featured in blue and the Gly-(I,S) proton pair shown in red. (**B**) Zoom of the LLC ^1^H spectrum of AlaGly dipeptide in the Gly-H^α^ region. Opposite-phase I and S are observed as ${Q}_{LLC}^{mol}$ is created. (**C**) Zoom of the ^1^H spectrum of AlaGly dipeptide in the Gly-H^α^ region ${Q}_{LLC^{\prime} }^{mol}$ is created. For (**B**,**C**) the method (“pulse sequence”) used to excite LLC’s is outlined, featuring the selective 180° pulse (grey-filled shape) used to invert I or S spins and the LLC evolution period, *τ*_*LLC*_.

The magnetic interactions of a pair of atomic nuclei with the outside bear the imprint of singlet and triplet functions whenever the *J*-coupling between the nuclear magnetic moments of the two atoms overcomes their couplings with other nuclei. Couples of atoms possessing nuclear spins, taken together, are perceived differently than isolated magnetic nuclei by structural neighbours. We treat herein the interactions of two coupled protons with angular momenta ½$\hslash$ in the molecular frameworks of a dipeptide and of a protein. The protons belonging to aliphatic glycine atoms Gly-H^α1,2^ in a dipeptide (Fig. 1) are noted I and S, respectively. There are two possible orientations, (*α*,*β*)_*I,S*_, for each of their magnetic moments with respect to an external magnetic field, ***B***_0_. The symbiotic character of two coupled spins that only interact with each other^13^ is described by the singlet-triplet wavefunctions, i.e., the nuclear spin-permutation antisymmetric singlet state, ${S}_{0}=N(|{\alpha }_{I}{\beta }_{S} > -|{\beta }_{I}{\alpha }_{S} > )$, and the three symmetric triplet states, ${T}_{+1}=|{\alpha }_{I}{\alpha }_{S} > ,{T}_{0}=N(|{\alpha }_{I}{\beta }_{S} > +|{\beta }_{I}{\alpha }_{S} > ),{T}_{-1}=|{\beta }_{I}{\beta }_{S} > ,$ with *N* = 2^−1/2^. The decays of singlet-state populations are the least perturbed by spin-permutation symmetric interactions, such as the dipole-dipole interaction between the two nuclei, making singlet-triplet transitions the nuclear-magnetism equivalents of electronic phosphorescence. Collective spin order with reduced sensitivity to dipolar interactions compared to classical spin-state populations can be excited based on the population differences between singlet and triplet states, provided that any external magnetic field are removed or eclipsed by strong radio-frequency irradiation^13,24^. In this spin order, the memory of initial magnetisation of the sample may persist for one hour and longer^13,25^.

Coherent superpositions between singlet and triplet states^21,26^, known as long-lived coherences (LLC’s) are:

1 $${Q}_{LLC}=|{S}_{0} > \, < {T}_{0}|+|{T}_{0} > \, < {S}_{0}|$$

The sensitivity of LLC’s to the presence of nearby nuclei can be expected to yield new information compared to classical coherences.

### Switching the sign of LLC’s

Expressing (1) in terms of Cartesian operators^27^, it can be readily seen that LLC are excited creating via guided magnetisation evolution (‘spin dynamics’) with user-applied radio-frequency pulses, starting from *I*_*z*_ − *S*_*z*_. This is performed using the pulse sequence shown in the inset of Fig. 1B, where the selective 180° pulse inverses H^α2^ (noted S) to transform the thermal-equilibrium Boltzmann distribution (*I*_*z*_ + *S*_*z*_) into a difference (*I*_*z*_ − *S*_*z*_) or H^α1^ (noted I) which leads to (−*I*_*z*_ + *S*_*z*_).

A further 90° pulse creates seed states ${Q}_{LLC,\,LLC\text{'}}^{s}\,$with components on the two spins antiparallel to each other and aligned with the direction of the radio-frequency irradiation field:

2 $${Q}_{LLC}^{s}={I}_{x}-{S}_{x}\,{\rm{and}}\,{Q}_{LLC\text{'}}^{s}=-{I}_{x}+{S}_{x}$$

which, in an isolated two-spin system, are transformed into the eigenstates they project on: $${Q}_{LLC}\,=$$$$[{I}_{x}-{S}_{x}+i({I}_{z}{S}_{y}-{I}_{y}{S}_{z})]$$ and $${Q}_{LLC\text{'}}=[-{I}_{x}+{S}_{x}+i(-{I}_{z}{S}_{y}+{I}_{y}{S}_{z})]$$, respectively. These two states, antisymmetric with respect to each other upon permutation of I and S, are energy-degenerate. This is in contrast with long-lived states (LLS)^24^, which remain identical when switching I and S.

When contributions of external nuclei *K*_*i*_ are accounted for, the magnetic eigenstates of the Gly molecular subsystem, ${Q}_{LLC}^{mol}$, will feature perturbations compared to those of an isolated (*I,S*) spin pair, ${Q}_{LLC}$:

3 $${Q}_{LLC}^{mol}={Q}_{LLC}+{\sum }^{}{F}_{i}(I,S,{R}_{i})=[{I}_{x}-{S}_{x}+i({I}_{y}{S}_{z}-{I}_{z}{S}_{y})]+\sum _{i,j}{F}_{i}^{j}(I,S,{K}_{i})$$

Interestingly, the closely-related state:

4 $${Q}_{LLC\text{'}}^{mol}={Q}_{LLC\text{'}}+{\sum }^{}{F}_{i}(I,S,{R}_{i})=[-({I}_{x}-{S}_{x})-i({I}_{y}{S}_{z}-{I}_{z}{S}_{y})]+\sum _{i,j}{F}_{i}^{j}(I,S,{K}_{i})$$

is no longer energy-degenerate, and no longer fully symmetric to the (I,S) permutation with respect to ${Q}_{LLC}^{mol}$.

The structure of the states in (3) and (4) including terms ${F}_{i}^{j}\,\,$is detailed in the Supporting Information. These states can be obtained by evolving, via spin-dynamics from equilibrium, ${Q}_{LLC}^{mol,s}=({I}_{x}-\,{S}_{x})+{K}_{x}$and ${Q}_{LLC\text{'}}^{mol,s}=(\,-\,{I}_{x}+{S}_{x})+{K}_{x}$, respectively.

${Q}_{LLC}^{mol}$ and ${Q}_{LLC\text{'}}^{mol}$ are eigenstates of the system with relaxation time constants very close to those of LLC’s in two-spin systems when the coupling between the two neighbouring spins *I* and *S* largely prevails over scalar couplings of nuclei *I* and *S* with external spins, *K*_*i*_, i.e., ${J}_{IS}\gg {J}_{I,S{K}_{i}}$. The term in square brackets in Eq. (3) is reminiscent of a two-spin LLC. This term is formed by reciprocally-opposed magnetisation components of the two *J*-coupled Gly-H^α^ nuclei, components which are sustained in the plane transverse to the external magnetic field, ***B***_0_, by a radio-frequency modulated field. The structure of the remaining components *F*_*i*_ depends on the values of the small ${J}_{I,S{K}_{i}}$ couplings external to the *I,S* spin pair (Supporting Information), terms that depend on the complex equilibrium between the different structures the peptide adopts in the solvent.

We treated the evolution of a spin system similar to the Gly part of the dipeptide (details in the Supporting Information) in a spin-evolution computation performed with Spinach^28^ and GAMMA libraries^29^. Considering the Gly amide proton as external spin ‘K’ and distances similar to those between the Gly-H^N^ and Gly-H^α^ protons in the minimized molecular conformation in Fig. 1A and ^3^*J*_I/S,K_ couplings that differ by 2 Hz, we calculated a contribution *F*(*I*,*S*,*K*) from the Gly-H^N^ (‘K’) spin to Eq. (3) using SpinDynamica^30^ simulation package (See Supporting Information).

It is noteworthy that the theoretical derivation of eigenstates given here considers a simplified system with 3 spins, only to explain qualitatively the origin of the effect. Real systems contain too many parameters to be considered in a Liouville diagonalization and Molecular Dynamics-derived distances and couplings should consider multiple conformations and dynamic equilibria effects, which is beyond the scope of this study. Switching the signs of I and S spins in *Q*_*LLC*_ results in a change of the overall sign of the operator due to the spin-exchange symmetry of LLC’s.

### Experimental results on the interactions of LLC’s with water-exchangeable protons

LLC’s evolve in time, oscillating at a frequency corresponding to the eigenvalue of the eigenstate described in Eq. (1) and they decay according to their auto-relaxation rate constants, *R*_*LLC*_ and ${R}_{LLC^{\prime} }$:

5 $$d{Q}_{LLC,LLC^{\prime} }^{mol}/dt=-({R}_{LLC,LLC^{\prime} }+2\pi i{\nu }_{LLC}){Q}_{LLC,LLC^{\prime} }^{mol}$$

where *v*_*LLC*_ is the oscillation frequency_._ The diagonalization of the full Liouvillian of a 3-spin system (I,S,K) shows that for small values of *J*-couplings to the outside spin K with respect to *J*_*IS*_, ${\nu }_{LLC}\approx {\nu }_{LLC\text{'}}\approx {J}_{IS}$ when *J*_*IS*_ is the dominant coupling for the spin system and *R*_*LLC*_ is the relaxation rate constant, which effectively describes the decay of the signal. Experimental methods whereby the oscillation and relaxation time is chosen as multiples of the LLC evolution period (*τ*_*LLC*_ = *n*/*v*_*LLC*_, with n integer) were used^31,32^. These methods enable us to derive the *R*_*LLC*_ relaxation time constant from the fit of an exponential decay, rather than fitting an oscillating function. This is a fast way of probing interactions, compared to time-consuming 2D spectroscopy, as the entire set of experiments for one of the experimental conditions takes less than three hours to record.

The relaxation rate constants of LLC’s are driven by dipolar interaction between coupled spins *I* and *S*, interactions with external relaxation sources, *K*_*i*_, and coherent effects. Both types of sources can increase the observed relaxation rate constants, as the eigenstates in Eq. (1) are altered by additional terms, *F*_*i*_, introduced by adding protonated water to the sample. The focus of this study is to identify the contribution of interactions with water-exchangeable protons to *R*_*LLC*_ and ${R}_{LLC^{\prime} }$ relaxation rates that can be used in a structural context. The complex relaxation mechanisms underlying this contribution will be detailed in a further study. We measured the impact of water-exchangeable H-N protons on LLC relaxation by increasing the protonated:deuterated water ratio in the sample. The sign of LLC’s with respect to water magnetization was switched between ${Q}_{LLC}^{mol}$ and ${Q}_{LLC^{\prime} }^{mol}$. This leads to nuclear magnetic configurations similar to those used in ‘optimised spectroscopy’ implementation^33^ and fast-acquisition spectroscopy^34,35^.

**Figure 2:**
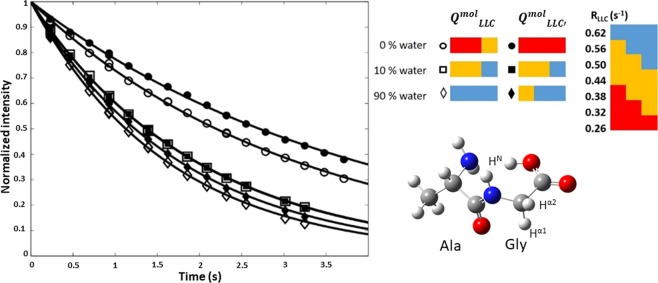
Observation of the effect of water-exchangeable protons on Gly-H^α^ LLC’s in the AlaGly dipeptide. The plot on the left shows the time dependence of ${Q}_{LLC}^{mol}$ (open symbols) and ${Q}_{LLC^{\prime} }^{mol}$ (full symbols) intensities as the H_2_O:D_2_O ratio in the sample is increased. Intensity errors are of the size of the symbols. The LLC relaxation rate enhancement effect is shown in colour coding on the right side, with blue indicating high relaxation enhancement and red indicating low relaxation rate constants.

In fully-deuterated solvent, we measured distinct decays for ${Q}_{LLC}^{mol}$ and ${Q}_{LLC^{\prime} }^{mol}$ (Fig. 2A). The pertaining relaxation rate constants, *R*_*LLC*_ = 0.32 and ${R}_{LLC^{\prime} }=0.25\pm 0.01{s}^{-1}$, show that, in the most-populated configuration of the AlaGly dipeptide, in fully-deuterated water, the ${Q}_{LLC^{\prime} }^{mol}$ configuration excited via (*S*_*x*_ − *I*_*x*_) is slower-relaxing than the ${Q}_{LLC}^{mol}$ configuration excited via (*I*_*x*_−*S*_*x*_). Therefore, a first observation is that the relaxation rate constants of long-lived coherences can be optimized by selecting the most favourable proton to selectively invert. In the case of AlaGly aliphatic coupled protons, $\,{Q}_{LLC^{\prime} }^{mol}$ has a relaxation time constant superior by 20% to that of ${Q}_{LLC}^{mol}$. The relaxation contributions to ${Q}_{LLC}^{mol}$ from outside the (*I,S*) pair are larger than contributions to ${Q}_{LLC^{\prime} }^{mol}$, in deuterated solvent.

Upon addition of protonated water up to a H_2_O:D_2_O ratio of 10%:90%, both ${Q}_{LLC}^{mol}$ and ${Q}_{LLC^{\prime} }^{mol}$ relaxation rate constants increase to ${R}_{LLC}\approx {R}_{LLC^{\prime} }=0.50\pm 0.01\,{s}^{-1}$. At 90% protonated water, the relaxation rate constants increase to ${R}_{LLC}\approx {R}_{LLC^{\prime} }=0.58\pm 0.01\,{s}^{-1}$. The titration of water protons in the dipeptide ensemble leads to a differential broadening of ${Q}_{LLC}^{mol}$ and ${Q}_{LLC^{\prime} }^{mol}$ lines. This occurs due to interactions with water protons that remain in the solvent as well as with the protons now appearing at exchangeable sites. The estimated direct solvent accessibility at the positions of Gly- Hα1 and Gly- Hα2 is similar, both sites being highly exposed. However, the contribution of external water to relaxation is estimated by numerical simulations to be small compared to intra-molecular interactions, when effective motional correlation times on the order of the ps are considered for the interaction. The loss of coherence mainly occurs as the aliphatic protons experience J-couplings with outside protons altering the structure of the LLC eigenstate. The variation of these couplings due to exchange, contribution known as scalar relaxation of the first kind^36^, will further contribute to relaxation. Both these contributions will increase relaxation rate constants with increasing values of the J-coupling. Experimentally, the relative changes in *R*_*LLC*_ and ${R}_{LLC^{\prime} }$ values upon H_2_O addition show that contributions of water-exchangeable protons to the relaxation of the ${Q}_{LLC}^{mol}$ and ${Q}_{LLC^{\prime} }^{mol}$ configurations are different. The presence of exchangeable protons enhances the relaxation rate constant of ${Q}_{LLC^{\prime} }^{mol}$ more than it does for ${Q}_{LLC}^{mol}$. This behaviour is consistent with computer simulations of the full magnetization evolution carried out on a system of two spins featuring different couplings to a third. The spin dynamics behaviour was simulated using GAMMA libraries^29^ and Spinach^28^ within a three-spin system (*I*,*S*,*K*) similar to the Gly-Hα1, Gly-Hα2, Gly-HN system, featuring (^3^J_SK_ − ^3^J_IK_)/^2^J_IS_ ≈ 0.1 (Supporting Information). Water-exchangeable protons in the terminal carboxyl group, N-terminal amine and the glycine amide intervene in the structure of eigenstates when LLC’s are excited. We only took into account the contributions to relaxation from Gly-HN protons, the closest to the site of aliphatic-protons where LLC’s were excited. Magnetisation evolution predict that the configuration wherein the magnetization of spins S, which feature the strongest coupling to external spins K, is excited pointing in the same spatial direction as water magnetisation, configuration accessed via ${Q}_{LLC^{\prime} }^{mol,s}$, suffers the highest variation of its relaxation rate constant upon interaction with the external spin (See Figure S1.3 and further discussion). The effect of the outside spin K on the *R*_*LLC*_ relaxation rate was found to be 10% smaller than on ${R}_{LLC^{\prime} }$. It was verified that most of the perturbation arises via coherent evolution, i.e., the perturbation via the coupling with the ‘K’ spin, rather than via dipolar interactions. This is consistent with experimental data in Fig. 2, where Gly-Hα1 corresponds to spin I and Gly-Hα2 to spin S. Therefore, the experimentally-observed behaviour of *R*_*LLC*_ and ${R}_{LLC^{\prime} }$ values, correlated with the expected enhancement in relaxation rates based on theoretical considerations and computer simulations, assigns I and S spins to Gly-Hα1 and Gly- Hα2, respectively. We verified the bijective correspondence between the positions of these hydrogens in the molecular structure with respect to Gly-HN and the positions of their signals in the 1D spectrum, which were the only information used to encode long-lived coherences with different signs (Gly-Hα1,Hα2) ↔ (I,S). Gly residues in proteins are adapted probes for interactions, especially in loops or intrinsically disordered proteins^37^ where LLC lifetimes are expected to be favourable. Other probes have been proposed, in addition to Gly aliphatic protons^38^.

**Figure 3:**
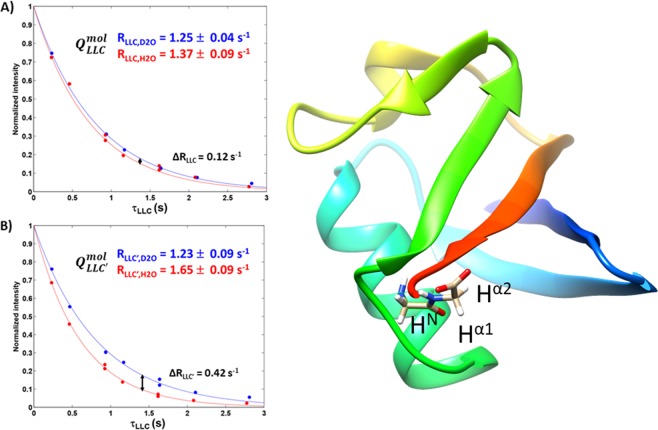
Observation of the effect of water-exchangeable protons on coherences derived from Gly-H^α^ based LLC’s in Ubiquitin. The plot on the left shows the time dependence of ${Q}_{LLC}^{mol}$ and the one on the right the dependence of ${Q}_{LLC^{\prime} }^{mol}$ as H_2_O is added to the solution. Intensity errors are of the size of the symbols.

We applied the same LLC symmetry-based method for the study of Ubiquitin, in a part of its structure where LLC lifetimes are sufficient to enable this type of observation, the C-terminus Gly-76 (Fig. 3). Again, marked variations between the behaviour of ${Q}_{LLC}^{mol}$ and ${Q}_{LLC^{\prime} }^{mol}$ states are observed between the samples in deuterated and protonated water. These variations occur in a relaxation-rate zone superior to that of AlaGly, as the number of outside neighbours and the overall tumbling time are larger in the protein. Simulations with Spinach^28^ show that the variation in the two diastereotopic protons is correlated with their positions with respect to the Gly-76 HN (Supporting Material). It was noted (Figure S1.6) that the state excited via a seed in which the more strongly coupled aliphatic proton has the same sign as its coupling partner, K, ${Q}_{LLC^{\prime} }^{mol,s}=-\,{I}_{x}+{S}_{x}+{K}_{x}$ ($I=H\alpha 1,\,S=H\alpha 2\,{\rm{and}}\,K=HN$) has a larger enhancement of the relaxation rate constant when transitioning from deuterated to protonated water. This behaviour is similar to the one observed in AlaGly.

We observed that, between the two coherent configurations based on Gly-H^α1^ and Gly-H^α2^, the presence of exchangeable protons will affect coherences excited via ${Q}_{LLC^{\prime} }^{mol,s}$ to a higher degree than those excited by ${Q}_{LLC}^{mol,s}$. This is because in ${Q}_{LLC^{\prime} }^{mol,s}$ the magnetic structure features positive magnetisation of the proton (here, Gly-H^α2^) that has a stronger *J*-coupling with the exchangeable site (here, Gly-H^N^). The structural information is obtained faster using long-lived coherences than using two-dimensional correlation-spectroscopy transfer via *J*-couplings^39,40^. 2D proton correlation spectroscopy takes sixteen hours to acquire in a 10%:90% protonated:deuterated water mixture, as the intensity of H^N^ signals is merely 5% that of the aliphatic protons, due to exchange broadening. LLC’s can provide structural information even in the absence of the signal of exchangeable peaks, as information is acquired on aliphatic hydrogens.

To summarise, we show that the lifetimes of long-lived coherences in a small peptide and the C-terminus of a protein are sensitive to interactions with water-exchangeable protons. Structural information on the proximity between aliphatic and water-exchanging protons in peptides and protein disordered loops, where Gly residues are frequent, can be obtained by switching the sign of the permutation-antisymmetric long-lived coherences with respect to water protons magnetisation. The new proposed NMR method requires no isotopic enrichment and only two 1D experiments of selectively created LLC’s are needed.

## Methods

The AlaGly dipeptide (70 mg, MW = 146.14 g.mol^−1^) with natural-abundance spin isotopes, purchased from SigmaAldrich (product A0878) was dissolved in D_2_O (1 ml), *D*_2_*O*:*H*_2_*O* = 90:10 and *D*_2_*O*:*H*_2_*O* = 10:90 solutions. The Ubiquitin experiment in deuterated water was conducted by dissolving 10 mg of UBQ (SigmaAdrich product code U6253) in 585 μL of D_2_O while UBQ experiment in *D*_2_*O*:*H*_2_*O* = 10:90 mixture was done by dissolving 15.1 mg UBQ in 540 μL H_2_O and 60 μL D_2_O (*C*_*M*_ = 2.93 *mM*. NMR spectra were recorded at *T* = 300 *K* on a Bruker Avance spectrometer operating at ***B***_0_ = 11.75 *T*, i.e., at the Larmor proton frequency *v*_0_ = 500.13 *MHz*, equipped with a 5-mm BBO BB/19F/^1^H/D probe. Spectral intensities were extracted using TopSpin. The dependences of spectral intensities on evolution delays were fitted using the dedicated Matlab function and errors were calculated from a Monte-Carlo analysis performed using 100 variations within the spectral noise level for each fit.

Proton reference 1D spectra and LLC experiments were recorded with 8 transients and a recovery delay of 1.82 s. The (H^α1^, H^α2^) pair of nuclei feature a ^2^*J*_IS_ coupling value *J*_*IS*_ = 17.2 *Hz* and a frequency difference of Δ*v*_*IS*_ = 55.7 *Hz* at the given ***B***_0_ value. To record an LLC’s experiment with the inversion of one Gly-H^α^, we used the pulse sequence describes in Fig. 1. The carrier frequency was placed in the middle of the two Gly-H^α^ doublets. In practice, LLC and LLC’ terms are excited by placing the transmitter frequency in the middle of Gly-H^α^ doublets, at ${\nu }_{av}=\frac{{\nu }_{I}+{\nu }_{S}}{2}=1820Hz$ and applying a selective 180° pulse at an offset Δ*v*_*RF*_ corresponding to the centre of the resonances of I or S. For the inversion of the I, Δ*v* = −27.85 *Hz*, and for the inversion of the Gly-S, Δ*v* = −27.85 *Hz*. The selective pulse is followed by a 90° with phase y pulse in order to reach the observable coherence ±(I_*x*_ − S_*x*_). Finally, a continuous wave radiation, **B**_1_(*t*), converts opposite-orientation vectors into *Q*_*LLC*_ or ${Q}_{LLC^{\prime} }$ (Fig. 1B,C). The intensity of the 90° hard pulse was *γ* ***B***_1_ = 14908 *Hz* and its duration was *τ*_90_ = 16.83 *μs*. LLC’s were sustained during variable delays *τ*_*LLC*_ using continuous-wave (c.w.) irradiation with a radio-frequency (RF) amplitude *v*_1_ = 2.5 *kHz*. The amplitude of the selective pulse at 180° was *γ****B***_1_ = 40.5*Hz* and its duration was *τ*(*p*11) = 30 *ms*.

## Supplementary information

Supporting Information for Water hydrogen uptake in biomolecules detected via nuclear magnetic phosphorescence
