# Supplementary material for: Water hydrogen uptake in biomolecules detected via nuclear magnetic phosphorescence
Source: Sci Rep. 2019 Nov 19;9:17118. doi: 10.1038/s41598-019-53558-8 (PMC6864387; doi:10.1038/s41598-019-53558-8)
Supplement: Supplementary file 1 — Supporting Information for Water hydrogen uptake in biomolecules detected via nuclear magnetic phosphorescence [file 41598_2019_53558_MOESM1_ESM.pdf]

Supporting Information

for

Water hydrogen uptake in biomolecules detected via nuclear magnetic phosphorescence

Aude Sadet[1], Cristina Stavarache[1,2], Florin Teleanu[1,3], and Paul R. Vasos[1,4]*

[1]Research Institute of the University of Bucharest (ICUB), 36-46 B-dul M. Kogalniceanu, RO-050107 Bucharest, Romania

[2]"C.D. Nenitescu" Centre of Organic Chemistry, 202-B Spl. Independentei, RO-060023 Bucharest, Romania

[3]"Universitatea Babes-Bolyai", Facultatea de Chimie si Inginerie Chimica, Strada Arany Janos 11, Cluj-Napoca, Romania

[4]"Horia Hulubei" National Institute for Physics and Nuclear Engineering IFIN-HH, Extreme Light Infrastructure - Nuclear Physics ELI-NP, 30 Reactorului Street, RO-077125 Bucharest-Magurele, Romania

Table of contents

SI1. Details on the spin dynamics simulations and molecular energy minimisation

- Long-lived coherences-related states in three-spin systems
- Molecular dynamics calculations and spin dynamics on specific molecular systems
- Spin dynamics for the AlaGly-like system
- Spin dynamics for the Ubiquitin-like system

SI2. Further experimental data detailing the temperature dependence of the exponential decay rates, their reproducibility, the effects of sustaining amplitude and suppression of water signals on the results.

SI1. Details on the spin dynamics simulations and molecular energy minimisation

Long-lived coherences-related states in three-spin systems

Considering a J-coupled two-spin system with spins denoted I and S, the separation between their Larmor frequencies ν_I and ν_S is $\Delta\nu_{IS} = \nu_I - \nu_S > 0$. The Hamiltonian of such system in the high-field approximation[1] is $H = \nu_I I_z + \nu_S S_z + J I_z S_z$ and the eigenbasis is known as Zeeman product basis $\Phi_{PB} = \{\alpha_I\alpha_S, \alpha_I\beta_S, \beta_I\alpha_S, \beta_I\beta_S\}$.

In order to generate an LLC, the pulse sequence discussed in the main text (outlined below) is used.

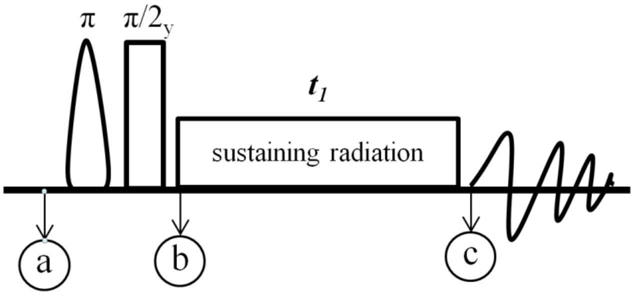

Figure S1.1 The Liouville states analysis in the current chapter concerns evolution between time points b and c in a spin system containing an (I,S) spin pair and an external coupling partner, K.

Starting from an initial equilibrium magnetization $\sigma_{eq} = I_z + S_z$, a selective 180° pulse rotates the magnetization of the S spin to generate $I_z - S_z$. Then, a hard 90° pulse rotates both operators around the y-axis to get a $I_x - S_x$ state that is sustained for a time t_1 via continuous-wave (cw) radio-frequency with an amplitude $|\nu_1| = |-\gamma B_1/2\pi| \gg \Delta\nu_{IS}$ and a rf carrier ν_{rf} placed at half-way between the two chemical shifts. These conditions are represented by the Hamiltonian $H = \nu_1(I_x + S_x) + J\vec{I}\cdot\vec{S}$. Due to its symmetry under spin-permutation operator, the new eigenbasis will be the singlet-triplet basis $\Phi_{STB} = \{T_{+1}, T_0, S_0, T_{-1}\}$. The transformation matrix between the two bases $\Phi_{PB} = V\cdot\Phi_{STB}$ where V is given by the equation (SI 1):

$$V = \{\{1,0,0,0\},\{0,\sqrt{1/2},\sqrt{1/2},0\},\{0,\sqrt{1/2},-\sqrt{1/2},0\},\{0,0,0,1\}\}. \qquad \text{(SI 1)}$$

Because the dominant quantisation axis is shifted from the $\mathbf{B}_0$ direction (z) to the rf-field direction (x) operators are transformed into a tilted frame via the following equation (SI 2):

$$\begin{pmatrix} K'_x \\ K'_y \\ K'_z \end{pmatrix} = \begin{bmatrix} cos\theta & 0 & -sin\theta \\ 0 & 1 & 0 \\ sin\theta & 0 & cos\theta \end{bmatrix} \begin{pmatrix} K_x \\ K_y \\ K_z \end{pmatrix} \qquad \text{(SI 2)}$$

where $\theta = arctan\left[\frac{\nu_1}{\Delta\nu_{I,S}}\right]$. As we already mentioned, $\nu_1 \gg \Delta\nu_{IS}$, so $\theta = 90°$. As a consequence, the corresponding state in the tilted frame will be $I'_z - S'_z = I_x - S_x$. This in turn may be written in the Φ_{STB} as a coherent superposition between the S_0 and the T_0 with the following structure: $Q_{LLC} = |T_0\rangle\langle S_0| + |S_0\rangle\langle T_0|$. The derivation is given in the following matrix transformation sequence (Table 1):

Product Basis Operator	Tilted-Frame Product-Basis Operator	Singlet-Triplet Basis Operator
$\frac{1}{2}\begin{pmatrix} 0 & -1 & 1 & 0 \\ -1 & 0 & 0 & 1 \\ 1 & 0 & 0 & -1 \\ 0 & 1 & -1 & 0 \end{pmatrix}$ $I_x - S_x$	$\begin{pmatrix} 0 & 0 & 0 & 0 \\ 0 & 1 & 0 & 0 \\ 0 & 0 & -1 & 0 \\ 0 & 0 & 0 & 0 \end{pmatrix}$ $I'_z - S'_z$	$\begin{pmatrix} 0 & 0 & 0 & 0 \\ 0 & 0 & 1 & 0 \\ 0 & 1 & 0 & 0 \\ 0 & 0 & 0 & 0 \end{pmatrix}$ $\vert T_0 >< S_0\vert + \vert S_0 >< T_0\vert$

Table S1: Matrix representation of the spin state before irradiation (left) and during the cw radio-frequency irradiation (tilted-frame) in the product basis (center) and singlet-triplet basis (right).

In order to evaluate the time evolution of the LLC (neglecting relaxation), one needs to solve the Liouville – von Neumann equation given by equation (SI 3):

$$\frac{d}{dt}\sigma(t) = -i[H, \sigma(t)] \qquad \text{(SI 3)}$$

where $\sigma(t)$ is the time-dependent density matrix of the system with $\sigma(0) = I_x - S_x$ and the aforementioned $H = \nu_1(I_x + S_x) + J\vec{I}\cdot\vec{S}$. As $(I_x + S_x)$ commutes with $\sigma(0)$, the only time-evolution contribution will arise from the *J*-coupling with $[\vec{I}\cdot\vec{S}, I_x - S_x] = i(2I_yS_z - 2I_zS_y)$. Noteworthy, the commutation relation for the symmetric state corresponding to the sum of transverse magnetization is $[\vec{I}\cdot\vec{S}, I_x + S_x] = 0.$ For a time-independent H, the solution has the following sandwich expression given in equation (SI 4):

$$\sigma(t) = exp(-iHt)\,\sigma(0)\,exp(iHt) \qquad \text{(SI 4)}$$

Substituting the exponential matrices, we obtain the time-evolution equation of the LLC (equation SI 5):

$$\sigma(t) = (I_x - S_x)\cos 2\pi Jt + (2I_yS_z - 2I_zS_y)\sin 2\pi Jt \qquad \text{(SI 5)}$$

Thus, the initial $I_x - S_x$ state will oscillate at a frequency given by the scalar coupling constant *J* between an in-phase and an anti-phase state. Adding the relaxation constant R_{LLC}, the evolution of the Q_{LLC} will be an oscillating exponential decay (equation SI 6):

$$\frac{d}{dt}Q_{LLC} = -[R_{LLC} + i(2\pi J)]Q_{LLC} \qquad \text{(SI 6)}$$

General considerations of long-lived coherence in three-spin systems

We considered the perturbation of the LLC states in the presence of a third spin *K* of the form:

$$Q_{LLC}^{mol} = Q_{LLC} + F(I, S, K) = \left[(I_x - S_x) + i\left(2I_yS_z - 2I_zS_y\right)\right] + F(I, S, K) \qquad \text{(SI 7)}$$

In order to identify the Q_{LLC}^{mol} state, one needs to calculate the eigenstates of the three-spin Liouvillian in the presence of cw radio-frequency pulse. To begin, the three-spin Hamiltonian is written as

$$H = \nu_1(I_x + S_x + K_x) + J_{IS}\vec{I}\cdot\vec{S} + J_{IK}\vec{I}\cdot\vec{K} + J_{SK}\vec{S}\cdot\vec{K} \qquad \text{(SI 8)}$$

The operator we are interested in is the commutation superoperator $L = [H,\ ]$. For the case of two-spin system, the LLC eigenstate has the form $\left[(I_x - S_x) + i\left(2I_yS_z - 2I_zS_y\right)\right]$ so it would not change its shape during coherent evolution. For the 3-spin system, we chose AlaGly-inspired values for coupling constants in order to diagonalise the Liouvillian and identify the eigenstates. We treat herein a three-spin system with the main coupling constant $J_{IS} = 17\ Hz$, while the other two were much smaller: $J_{IK} = 1\ Hz$ and $J_{SK} = 3\ Hz$.

The dimensionality of the Liouvillian is $4^3 = 64$ so a total of 64 eigenstates were produced. The two-spin LLC state was projected onto these and those with the biggest projection coefficient were selected. Below, we list the eigenstates that resemble the original two-spin LLC state the most. All calculations were done using the SpinDynamica package[3].

If the scalar coupling constants to the third spin are equal ($J_{IS} = 17\ Hz$, $J_{IK} = 3\ Hz$ and $J_{SK} = 3\ Hz$), we get two quasidegenerate states which includes the classical two spin LLC terms. Rounded to two digits, the states are as follows

$$Q_{LLC}^{mol} = \frac{1}{4}\{[I_x - S_x + i\left(2I_yS_z - 2I_zS_y\right)] + \textstyle\sum_j F^j\,(I,S,K)\} \qquad \text{(SI 9)}$$

$$Q_{LLC'}^{mol} = \frac{1}{4}\left\{\left[-I_x + S_x + i\left(-2I_yS_z + 2I_zS_y\right)\right] + \textstyle\sum_j F^j\,(I,S,K)\right\} \qquad \text{(SI 10)}$$

For this case, we identified two terms $F^j, j = 1{,}2$ with amplitude greater than 0.1:

$$F^1(I,S,K) = 2I_xK_x - 2S_xK_x - i(4I_yS_z - 4I_zS_y)K_x$$

and

$$F^2(I,S,K) = \frac{1}{6}[-2I_yK_y - 2I_zK_z + 2S_yK_y + 2S_zK_z + i\left(2I_yK_z - 2I_zK_y - 2S_yK_z + 2S_zK_y\right) + \\ +4I_xS_yK_y + 4I_xS_zK_z - 4I_yS_xK_y - 4I_zS_xK_z + i(4I_xS_zK_y - 4I_xS_yK_z + 4I_yS_xK_z - 4I_zS_xK_y)]$$

In the limit $J_{IK} = J_{SK} = 0$ the two eigenstates are energy-degenerate and lack the $F^2(I,S,K)$ perturbation terms.

Molecular dynamics calculations and spin dynamics on specific molecular systems

Our approach was to simulate proton-only spin-systems that resemble those studied experimentally in the case of AlaGly and Ubiquitin (Ubq). In the paper, we described the different evolution of molecular LLCs when the two strongly coupled protons are selectively inverted. The presence (in H_2O) or absence (in D_2O) of residual scalar coupling with a third proton, the closest amidic proton, makes these two spin states evolve differently and a theoretical interpretation of this phenomenon is given in the following.

In order to define realistic distances for dipolar interactions, we performed simulation of the spin systems involved trying to replicate the actual configuration of the protons. A geometry optimization of the dipeptide using Gaussian 09[2] was performed at the DFT level of theory employing the PBE0 functional along with the Karlsruhe valence triple-zeta basis set with polarization (DEF2-TZVP). The frequency analysis indicated that the optimized structure is a true minimum on the potential-energy surface (Figure S1.2). The coordinates of the Ubq's Gly 76 residue and the neighbouring amidic proton (H-N) were extracted from the Protein Database entry 1UBQ.

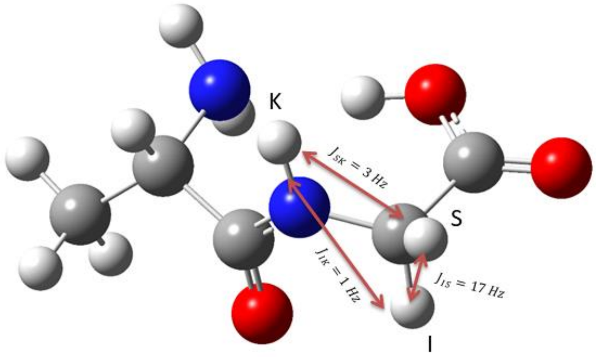

Figure S1.2: The optimized structure of AlaGly with highlighted protons considered for the molecular LLC; the geometry was optimized using DFT level of theory (PBE0/Def2TZVP) while the scalar coupling constants were selected close to the experimental ones.

Calculated atomic coordinates in the AlaGly structure:

N	0.28296600	-0.83712300	1.27221200
H	-0.32832600	-1.30845100	1.92731800
C	0.55270700	-1.67465800	0.09340600
H	0.80611800	-2.67467200	0.46194200
C	-0.57612100	-1.76012800	-0.90892900
C	1.80761900	-1.02939500	-0.48985800
H	-0.78461700	-0.77264200	-1.32469000
H	-0.31302800	-2.41757900	-1.74029800
H	-1.48028300	-2.14822900	-0.43483200
O	1.76948700	-0.21702700	-1.38623700
N	2.94253800	-1.33622500	0.20164700
C	4.02284500	-0.39419100	0.30032800
C	3.92297500	0.56667600	1.48561100
H	4.98736200	-0.89787700	0.37084900
H	4.03055900	0.20897900	-0.60931200
O	2.77231600	0.60937600	2.15731100
O	4.85323200	1.25521800	1.79055900
H	2.83768100	-1.98520800	0.96487900
H	-0.18940100	0.01315500	0.98177800
H	2.07045900	0.01854000	1.80820300

Spin dynamics for the AlaGly-like system

When changing the coupling constants to $J_{IS} = 17\,Hz$, $J_{IK} = 1\,Hz$ and $J_{SK} = 3\,Hz$ in order to emulate the actual spin environment of the AlaGly system, the operators in the expressions SI 9 and SI 10 gain slightly different coefficients (for example I_x has a coefficient of 0.242 and S_x has a coefficient of 0.249) which enhance the distinction between Q_{LLC}^{mol} and $Q_{LLC'}^{mol}$. The degeneracy of the two eigenstates of the coherent Liouvillian is lifted when the scalar couplings to an outside spin K are different.

Simulations using the Spinach[4] package were performed in order to assess the evolution of molecular LLC's.

Two different simulations were performed: the first one where only the Gly protons were taken in consideration and the initial states (seed states) which excites LLC's were chosen as $Q_{LLC}^{s} = I_x - S_x$ and $Q_{LLC'}^{s} = -I_x + S_x$. The second one where the amidic proton was added started from $Q_{LLC}^{mol,s} =$

$I_x - S_x + K_x$ and $Q_{LLC'}^{mol,s} = -I_x + S_x + K_x$. The latter resemble the case of AlaGly dissolved in water, while the former mimics the behaviour of an isolated LLC similar to the case of AlaGly dissolved in D_2O. A correlation time of 0.05 ns was assumed and the Bloch-Wangness-Redfield model of relaxation was employed. As all spins are assumed to have the same chemical shift in the mixing period due to the radio-frequency (rf) irradiation, the coherent evolution consisted only in the *J*-coupling operators. The following coupling constants were used: $J_{IS} = 17\,Hz,\ J_{IK} = 1\,Hz,\ J_{SK} = 3\,Hz$. The time-dependent evolution of the molecular LLC was projected onto the initial state in order to evaluate its relaxation properties. In Figure S1.3, the different trajectories of both classical two-spin and three-spin LLC's are presented along with fitted relaxation rate constant.

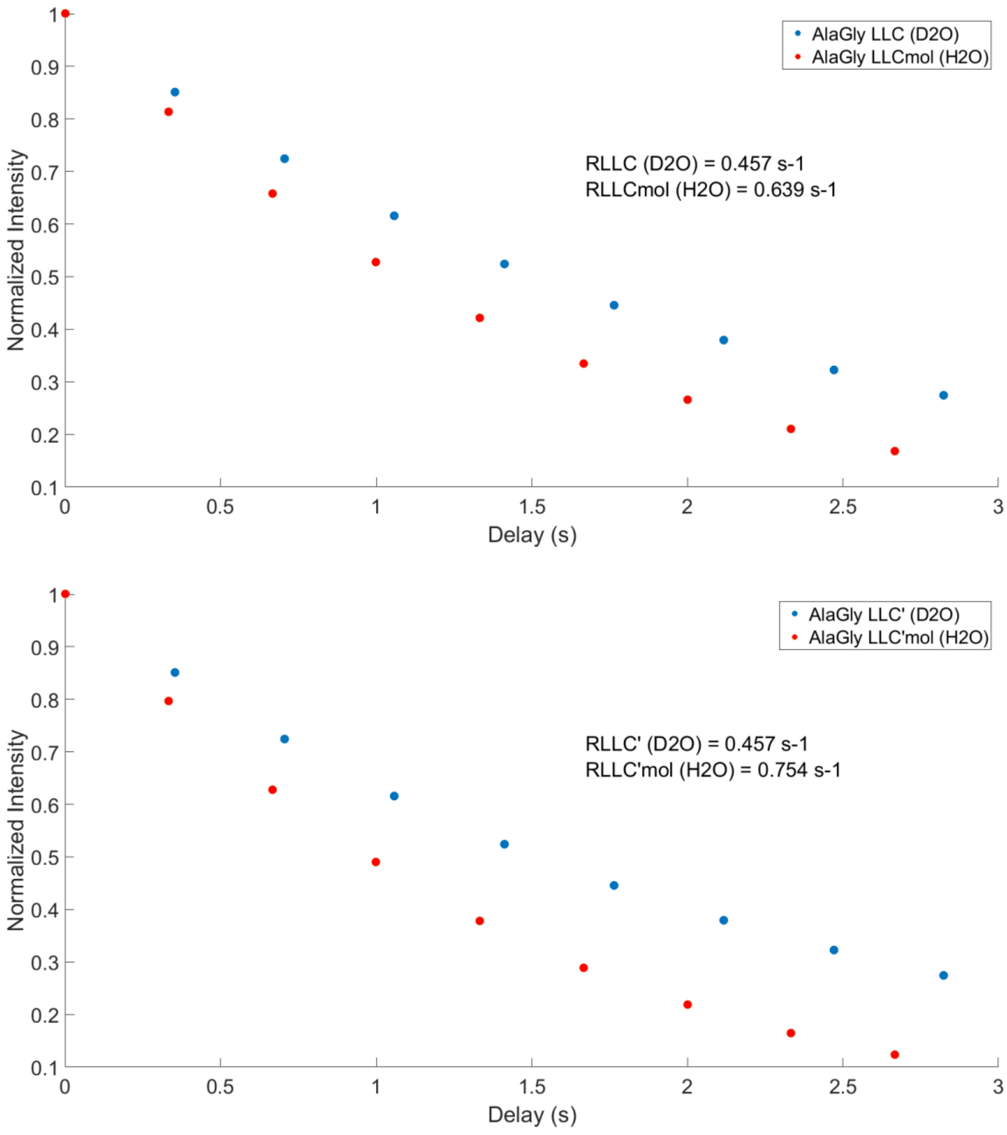

Figure S1.3: Time evolution of the long-lived coherences in an AlaGly-like spin-systems using the Spinach package in the two scenarios described above. Top figure: the evolution of $Q_{LLC}^{s} = I_x - S_x$ (D_2O case in blue) and $Q_{LLC}^{mol,s} = I_x - S_x + K_x$ (H_2O case in red); Lower figure: the evolution of $Q_{LLC'}^{s} = -I_x + S_x$ (D_2O case in blue) and $Q_{LLC'}^{mol,s} = -I_x + S_x + K_x$ (H_2O case in red);

For the case where only two protons are considered, the $I_x - S_x$ and $-I_x + S_x$ states behave the same as there is no asymmetry between them. Their relaxation rate constants are $R_{LLC} = R_{LLC'} = 0.457 s^{-1}$.

Upon insertion of a third spin, the amidic proton, these states gain further terms (as showed above) that render them inequivalent and, consequently, they have different relaxation rates. Starting from the $Q_{LLC}^{mol,s} = I_x - S_x + K_x$, respectively $Q_{LLC'}^{mol,s} = -I_x + S_x + K_x$, and projecting the overall evolution onto the initial state, the decay profiles are described above in red with different relaxation rate constant $R_{LLC_{mol}} = 0.639\ s^{-1}$, respectively $R_{LLC'_{mol}} = 0.754\ s^{-1}$. Thus, our simulations reproduce the presented empirical phenomenon of distinct relaxation rates for the two molecular long-lived coherences.

It is readily noted that the state in which the S spin is parallel to the K spin relaxes faster. Inversion of the couplings constants to $J_{IK} = 3\ Hz,\ J_{SK} = 1\ Hz$ determines the inversion of the relaxation rate constants $R_{LLC_{mol}} > R_{LLC'_{mol}}$. These results suggest that the relative values of residual scalar couplings dictate the overall relaxation profile of the states providing a method of discrimination between molecular LLC's.

We also conducted a simulation where we placed the K spin (the amide proton) on the perpendicular bisector of the segment connecting the I and S spins (the Gly H^{a1}, H^{a2} protons). The distance between the I and S protons was set to 2 A while the K spin was placed at 2 A apart from the middle point of line connecting the aliphatic protons. This setup renders the dipolar couplings equal for the spin pairs *(I,K)* and *(S,K)*. When using the following scalar coupling constants $J_{IS} = 17\ Hz,\ J_{IK} = 1\ Hz,\ J_{SK} = 3\ Hz$, we noted the same difference as in the case of our previous simulation (Figure S1.4). Thus, the LLC experiment can be used to detect differences in the scalar coupling of a spin-pair with an external one.

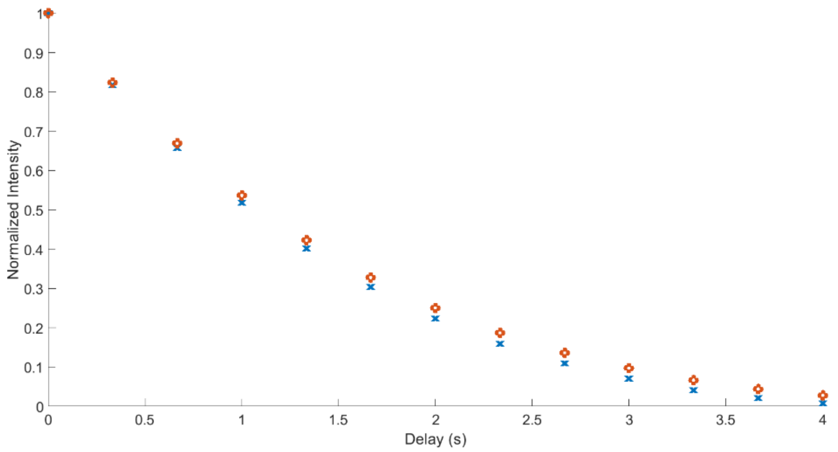

Figure S1.4: Time evolution of the $Q_{LLC}^{mol,s} = I_x - S_x + K_x$ (red dots) and $Q_{LLC'}^{mol,s} = -I_x + S_x + K_x$ blue crosses) in a three spins system with an isosceles triangle configuration; the K spin is placed on the perpendicular bisector of the segment connecting the I and S spins (2 A apart), at a distance of 2 A from the centre. Rendering the dipolar couplings equivalent still exhibits different evolutions for the two LLCs when the scalar coupling constants are different.

Spin simulation for the Ubiquitin-like system

For the Ubiquitin simulation, the coordinates of the aliphatic protons of the Gly76 residue and the closest amidic proton were extracted from the 1UBQ[5] entry of the Protein Data Bank. The rotational correlation time of Ubq is $\tau_c = 5\,ns$, as the molecular weight increases from the case of AlaGly but we considered a correlation time set to 1.5 ns to take into account the mobility of the C-terminus loop. Also, no anisotropy of rotational diffusion was considered. The geometry of the proton system considered is displayed below (Figure S1.5) and time-evolutions of the molecular LLCs are displayed in Figure S1.6 along with fitted relaxation rate constants.

The same pattern emerges as there is a different enhancement of the relaxation rate constant for the two molecular long-lived coherence. Again, the state in which the more strongly coupled spin ($J_{SK} = 3\,Hz > J_{IK} = 1\,Hz$) is parallel to the third spin $Q_{LLC}^{mol,s} = I_x - S_x + K_x$ vs $Q_{LLC'}^{mol,s} = -I_x + S_x + K_x$) has a bigger overall relaxation rate constant $R_{LLC'} = 3.050\,s^{-1} > R_{LLC} = 2.677\,s^{-1}$, similar pattern to the empirical data.

The degeneracy of LLC's in a J-coupled (I,S) system in various molecules is lifted by the introduction of a third coupled spin. The relaxation rate constants of LLC_{mol} and LLC'_{mol} diverge, as different additional relaxation contributions arise for the two, allowing to infer the positions of protons (I,S) from structural considerations.

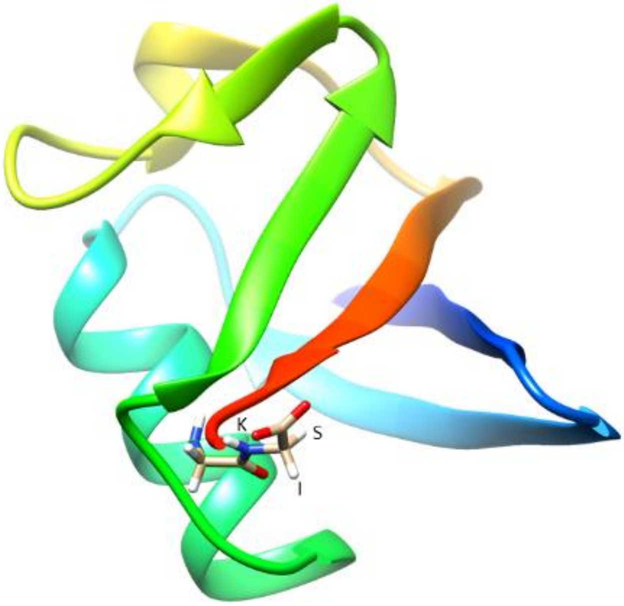

Figure S1.5: Highlighted Gly76 and Gly75 residues (wireframe) in Ubiquitin with assigned protons used in simulation (image rendered with Chimera software[6])

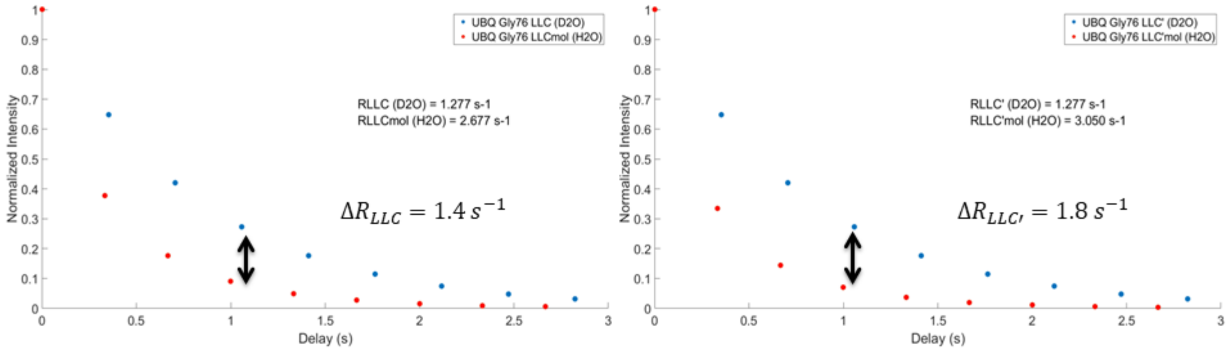

Figure S1.6: Time evolution of the long-lived coherences in Gly76-like spin-systems using the Spinach package in the two scenarios described above. Top figure: the evolution of $Q^{s}_{LLC} - S_x$ (D_2O case in blue) and $Q^{mol,s}_{LLC} = I_x - S_x + K_x$ (H_2O case in red); Lower figure: the evolution of $Q^{s}_{LLC} = -I_x + S_x$ (D_2O case in blue) and $Q^{mol,s}_{LLC'} = -I_x + S_x + K_x$ (H_2O case in red);

SI2 Further experimental data detailing temperature dependence, reproducibility, effects of sustaining amplitude and water suppression on the results

Data reproducibility

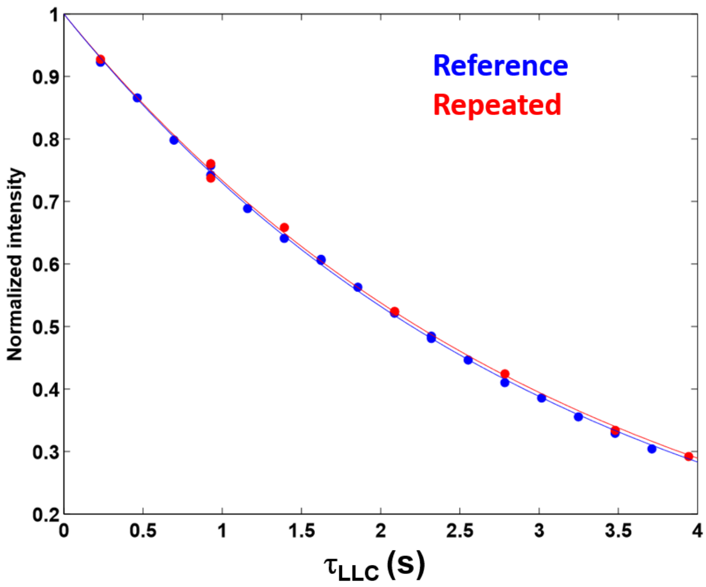

Fig S2.1: Data repeatability. Same decay (LLC in AlaGly) repeated several weeks apart.

LLC and LLC' data at higher temperature

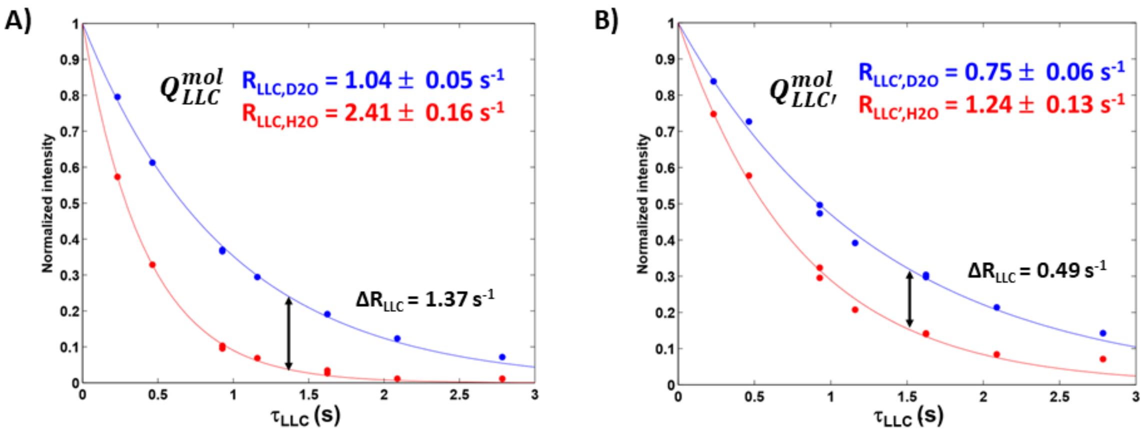

Fig S2.2: LLC and LLC' decays in Ubiquitin dissolved in D_2O and H_2O at T = 35°C, a different temperature than the results reported in the main text (T = 27°C). It is apparent that both LLC and LLC' states undergo more dramatic changes upon dissolution in water at this higher temperature.

Sustaining amplitude effects

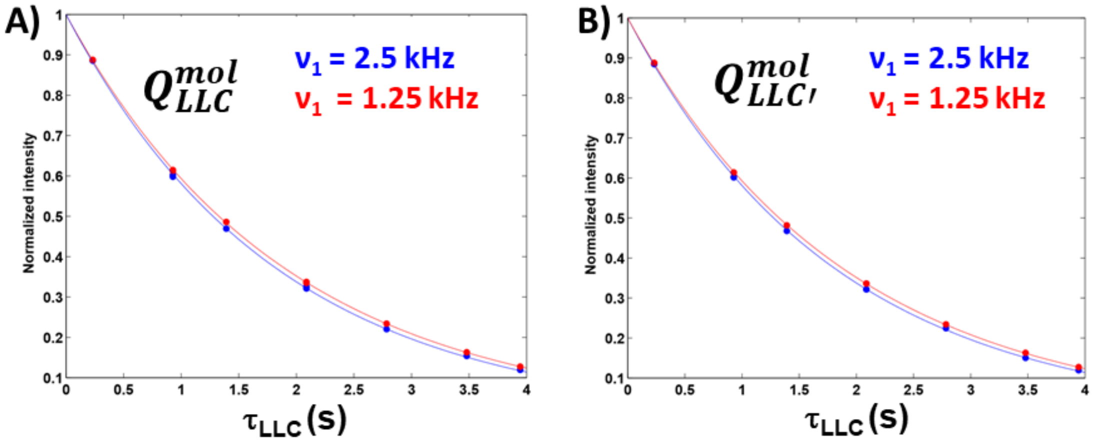

Fig S2.3: Comparison between LLC and LLC' data acquired with different pulse amplitudes on AlaGly at 27°C. The observed differences are minimal, even though the sustaining amplitude was halved.

Water suppression

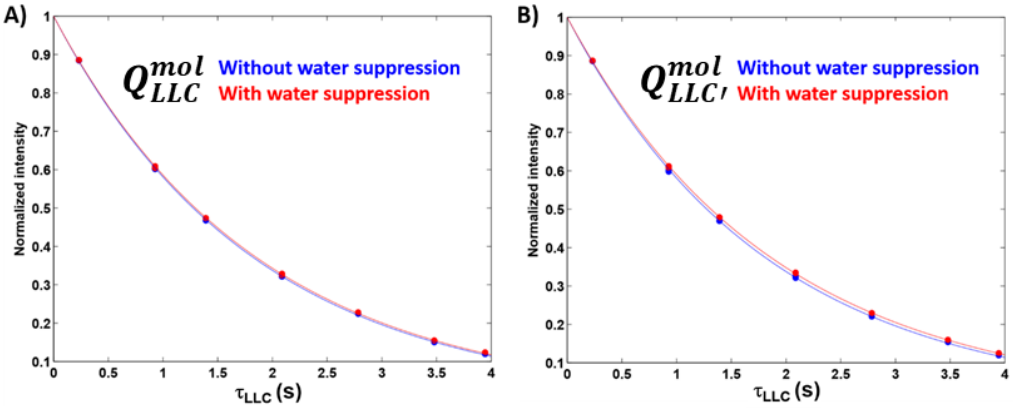

Fig S2.4 Comparison between water-suppression data and data recorded with no water suppression for LLC and LLC' in AlaGly at T = 27°C. Water suppression is based on the 'Watergate' spin echo scheme with pulsed field gradients placed after the LLC evolution period[7], and therefore was expected to show no interference with the detected results.

References

1 J. Cavanagh, W. J. Fairbrother, A. G. Palmer, M. Rance, N. J. Skelton in "Protein NMR Spectroscopy" (Academic Press 2006)

2 Gaussian 09, Revision E.01, M. J. Frisch, G. W. Trucks, H. B. Schlegel, G. E. Scuseria, M. A. Robb, J. R. Cheeseman, G. Scalmani, V. Barone, G. A. Petersson, H. Nakatsuji, X. Li, M. Caricato, A. Marenich, J. Bloino, B. G. Janesko, R. Gomperts, B. Mennucci, H. P. Hratchian, J. V. Ortiz, A. F. Izmaylov, J. L. Sonnenberg, D. Williams-Young, F. Ding, F. Lipparini, F. Egidi, J. Goings, B. Peng, A. Petrone, T. Henderson, D. Ranasinghe, V. G. Zakrzewski, J. Gao, N. Rega, G. Zheng, W. Liang, M. Hada, M. Ehara, K. Toyota, R. Fukuda, J. Hasegawa, M. Ishida, T. Nakajima, Y. Honda, O. Kitao, H. Nakai, T. Vreven, K. Throssell, J. A. Montgomery, Jr., J. E. Peralta, F. Ogliaro, M. Bearpark, J. J. Heyd, E. Brothers, K. N. Kudin, V. N. Staroverov, T. Keith, R. Kobayashi, J. Normand, K. Raghavachari, A. Rendell, J. C. Burant, S. S. Iyengar, J. Tomasi, M. Cossi, J. M. Millam, M. Klene, C. Adamo, R. Cammi, J. W. Ochterski, R. L. Martin, K. Morokuma, O. Farkas, J. B. Foresman, and D. J. Fox, Gaussian, Inc., Wallingford CT, 2016.

3 SpinDynamica code for Mathematica, programmed by Malcolm H. Levitt, with contributions by Jyrki Rantaharju, Andreas Brinkmann, and Soumya Singha Roy, available at www.spindynamica.soton.ac.uk.

4 H.J. Hogben, M. Krzystyniak, G.T.P. Charnock, P.J. Hore, I. Kuprov, "*Spinach - a software library for simulation of spin dynamics in large spin systems*", Journal of Magnetic Resonance, 208 (**2011**) 179-194.

5 PDB ID: 1UBQ

S. Vijay-Kumar, C.E. Bugg, W.J. Cook, "*Structure of ubiquitin refined at 1.8 A resolution*", Journal of Molecular Biology, 194 **(1987)** 531-544.

6 E.F Pettersen, T.D. Goddard, C.C. Huang, G.S. Couch, D.M. Greenblatt, E.C. Meng, T.E. Ferrin, "*UCSF Chimera--a visualization system for exploratory research and analysis*", Journal of Computational Chemistry, 13 **(2004)** 1605-1612.

7 M. Piotto, V. Saudek & V. Sklenar, "Gradient-tailored excitation for single-quantum NMR spectroscopy of aqueous solutions", Journal of Biomolecular NMR, 2 **(1992)** 661 – 666.
